# Peripersonal space plasticity in Schizophrenia: a motor training

**DOI:** 10.1192/j.eurpsy.2022.796

**Published:** 2022-09-01

**Authors:** F. Magnani, F. Ferroni, F. Ferri, M. Ardizzi, N. Langiulli, F. Giustozzi, F. Rasmi, R. Volpe, V. Lucarini, C. Marchesi, V. Gallese, M. Tonna

**Affiliations:** 1 University of Parma, Department Of Neuroscience, Psychiatric Unit, Parma, Italy; 2 University of Parma, Department Of Medicine & Surgery, Unit Of Neuroscience, Parma, Italy; 3 University G. D’Annunzio, Chieti, Department Of Neuroscience, Imaging And Clinical Science, chieti, Italy; 4 Université de Paris, Équipe Physiopathologie Des Maladies Psychiatriques, Umr 1266 Ipnp Inserm, Paris, France; 5 University of Parma, Department Of Medicine & Surgery, Unit Of Neuroscience, Psychiatric Unit, Parma, Italy; 6 Humboldt-Universit¨at zu Berlin, Berlin School Of Mind And Brain, Berlin, Germany

**Keywords:** schizophrénia, motor training, Psychosis, peripersonal space

## Abstract

**Introduction:**

A primary disruption of the bodily self is considered a core feature of schizophrenia patients (SCZ). The “disembodied” self would be underpinned by an inefficient body-related multisensory integration mechanism occurring in the Peripersonal Space (PPS). PPS is a plastic sector of space surrounding our body, whose extent is altered in SCZ. Although PPS represents a malleable interface marking the perceptual border between self and others, no study has investigated the potential alteration of its plasticity in SCZ.

**Objectives:**

We investigated the PPS extension and its plasticity in SCZ and their potential correlations with the clinical scales.

**Methods:**

Thirty SCZ and thirty healthy controls (HC) underwent a multisensory task to estimate PPS boundary before and after a motor training. Patients were also administered the Positive And Negative Syndrome Scale (PANSS) and the Examination of Anomalous Self-Experience (EASE).

**Results:**

Data confirm a narrower PPS extent in SCZ than in HC, whereas no differences in PPS expansion was found in the two groups after the motor training (Figure 1). Positive symptoms were associated directly with PPS extent and inversely with PPS plasticity. No associations were found between PPS and EASE domains. Figure1: Graphical representation of PPS expansion in SCZ and HC. Both panels show individual normalized sigmoid fits

**Figure:**
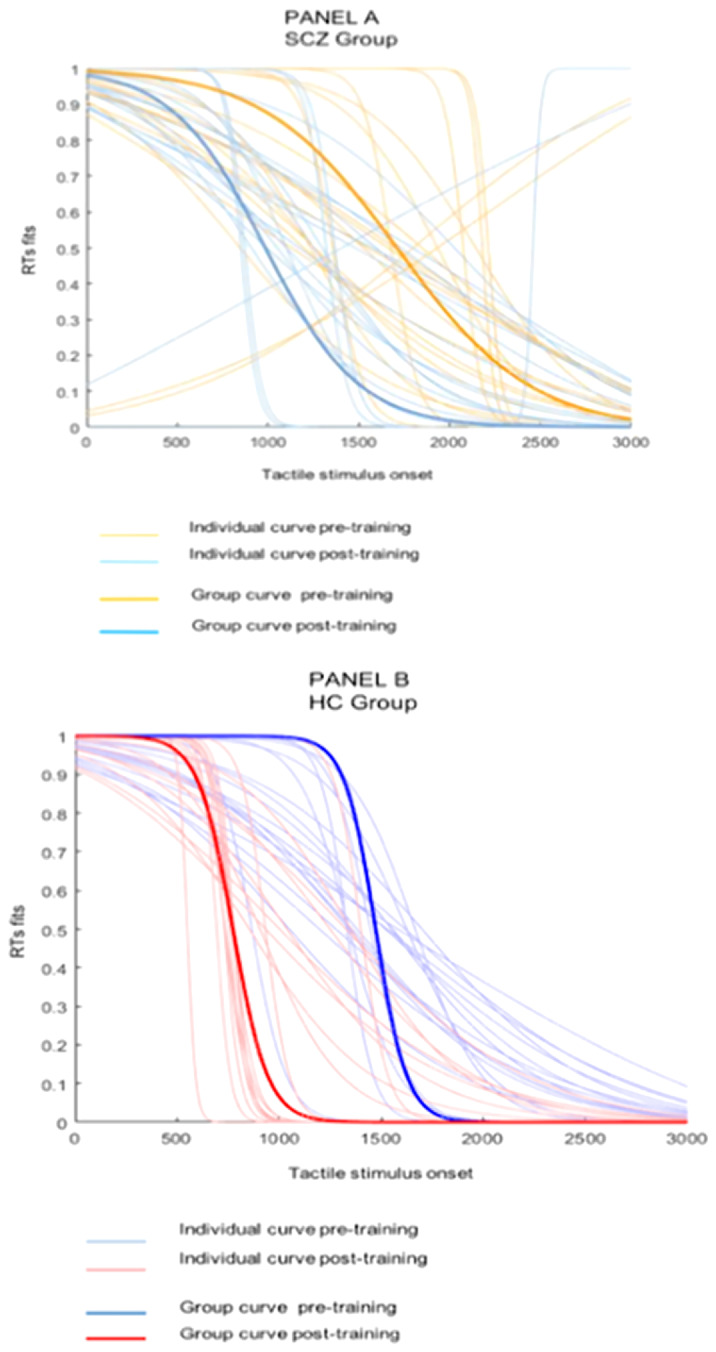

**Conclusions:**

The present study suggests a narrower PPS extent and a preserved PPS plasticity in SCZ with respect to HC. Both PPS extent and plasticity are related to the severity of positive symptoms. These results highlight the potential role of rehabilitation interventions in order to improve patients’ weakened body boundary.

**Disclosure:**

No significant relationships.

